# Losing fat, gaining treatments: the use of biomedicine as a cure for folk illnesses in the Andes

**DOI:** 10.1186/1746-4269-10-52

**Published:** 2014-07-03

**Authors:** Amy Blaisdell, Cecilie Vindal Ødegaard

**Affiliations:** 1 Department of Health Promotion and Development, Faculty of Psychology, University of Bergen, Christies Gate 13, 5020 Bergen, Norway; 2 Department of Social Anthropology, Faculty of Social Sciences, University of Bergen, Fosswinckelsgate 6, 5007 Bergen, Norway

**Keywords:** Peru, Andes, Biomedicine, Pharmaceuticals, Ethnomedicine, Folk illnesses, *Kharisiri*, Market vendors

## Abstract

**Background:**

This article explores how people in the Andes incorporate beliefs from both biomedical and ethnomedical systems in treating folk illnesses that often involve spiritual beings. The article focuses on the *kharisiri*—one who is believed to steal fat and blood from unsuspecting humans to make exchanges with the devil. The *kharisiri* in turn is rewarded with good fortune. Victims of *kharisiris,* however, fall ill and may die if untreated. Historically, *kharisiri* victims relied on ethnomedicine for treatment, but it appears biomedical pills are now perceived by some as an effective treatment. By drawing on participants’ attitudes towards biomedicine, and how people in the Andes conceptualize health, this article theorizes as to why biomedical pills are sought to treat *kharisiri* attacks but not for other folk illnesses.

**Methods:**

Fieldwork was conducted in Arequipa and Yunguyo among market vendors, who make up a significant portion of Peru’s working population. This type of work increases the risk of different illnesses due to work conditions like exposure to extreme temperatures, long-distance travel, and social dynamics. Biomedical and ethnomedical products are often sold in and around marketplaces, making vendors a compelling group for exploring issues relating to treatment systems. Qualitative data was collected in 2011 with a follow-up visit in 2013. Participant observation, informal conversations, and unstructured interviews with 29 participants informed the study.

**Results:**

Participants unanimously reported that biomedical pills are not capable of treating folk illnesses such as *susto* and *mal de ojo*. Several participants reported that pharmaceutical pills can cure *kharisiri* victims.

**Conclusions:**

In comparison to other folk illnesses that involve spiritual beings, those who fall ill from a *kharisiri* attack lose physical elements (fat and blood) rather than their soul (*ánimo*) or becoming ill due to a misbalance in reciprocal relations—either with humans or non-human beings such as *Pachamama*. Because the *kharisiri* is typically a stranger to the victim, the Andean concept of reciprocity appears to be irrelevant in terms of preventing and treating attacks. The association between *kharisiris*, biomedicine, and exploitation may also play a role in the use of biomedical pills.

## Background

Researchers have illustrated how people in the Andes perceive health and well-being as connected to their relationship with the animated natural surroundings and the spirit world (see e.g. [1-11]). Andean ethnomedical healing practices draw strongly on this understanding of the animated surroundings, as ritual offerings are made to *Pachamama* (*La Madre Tierra*, or Mother Earth) and other spiritual beings to uphold reciprocal balances that are central to well-being [8,12]. *Pachamama*, a feminine deity synonymous with the landscape and cycles of reproduction in agriculture and business, is viewed as the creator of the world and protector of life [4,10,13]. She is thought to prevent evil and illness, ensure profitable work, and a secure home [4]. To make certain that *Pachamama* provides for and protects humans, people in the Andes give her offerings to maintain reciprocity on a regular—sometimes daily—basis to show their gratitude. Bastien quotes a key informant in the Andes, “I am the same as the mountain, *Pachamama. Pachamama* has fluids which flow through her, and I have fluids which flow through me. *Pachamama* takes care of my body, and I must give food and drink to *Pachamama*[13].

If offerings are not made to *Pachamama*, it is thought that the earth can become “angry” and wreak havoc on those who fail to repay her generosity. This imbalance can be the source of cold winds, rapid temperature changes, or loud noises—conditions that people in the Andes believe can cause illness. Likewise, the *kharisiri—*a being who steals the blood and fat of humans—is said to “*hablar con el diablo*” (speak with the devil), which is also tied to the act of making payments through some kind of anti-social reciprocal exchange. By giving the devil blood and fat, the *kharisiri* helps to satisfy the devil’s cravings. In return, the devil rewards the *kharisiri* with good fortune at the expense of the victim, who may die if left untreated [4,14,15]. The *kharisiri* figure is known by different names in the Andes. *Kharisiri* is the term used by speakers of Aymara in South-Eastern Peru and Bolivia, while *nakaq* is the term generally used among speakers of Quechua. *Pishtaco* is the term used throughout Spanish-speaking Peru, especially in urban areas, as well as by Quechua-speakers of central Peru [16]. Some of the participants in this study used the terms *kharisiri* and *pishtaco* interchangeably, while others made a distinction; *kharisiris* make payments to the devil, while the *pichtaco* is a knife-using assassin.

Symptoms of an attack include pain in the abdomen where the bodily substances are extracted [14,17]. Another indication of an attack is seeing a black animal—typically a dog—around the time of the suspected attack, since *kharisiris* are understood as being able to transform from human beings into black animals. Other physical symptoms include fatigue, fever, headache, and vomiting^a^[2]. Typical symptoms of illnesses that involve spiritual beings more generally are loss of energy and appetite, headaches, depression, anxiety, seizers, and disturbed sleep patterns [2,12,18].

When one is suspected of falling ill due to an illness that involves spiritual beings, as a result of an imbalance in reciprocity—with *Pachamama* or within one’s social circle—traditional methods such as using medicinal herbs, drinking teas or homemade brews, holding ceremonies with traditional healers, and making ritual offerings are generally relied on to restore health. The restoration of health for *kharisiri* victims, in contrast, has been known to involve two possibilities; either to kill the *kharisiri*, or to replace lost substance with a similar substance [17]. But in Yunguyo, a small border town high in the Andes on the shores of Lake Titicaca, the authors’ research reveals that people are seeking biomedical pills (also to be referred to in this article as pharmaceutical pills, or simply, pills) to cure *kharisiri* attacks. This case is significant in that biomedical pills were not viewed by this study’s participants as capable of curing other folk illnesses that often involve spiritual beings.

This article theorizes as to why this is so by exploring the differences between how the *kharisiri* is conceptualized, mainly in comparison to *susto* (fright sickness or soul loss) and *mal de ojo* (evil eye). We will explore participants’ understanding of biomedicine, and then how they apply this understanding to several folk illnesses that involve spiritual beings. We will then discuss why it is that a “cross-over”—the use of treatment from one medical system to treat a sickness typically belonging to another—may be occurring in the case of the *kharisiri*. The initial idea for this article developed as the second author learned about the use of biomedical pills to cure *kharisiri* attacks during fieldwork conducted in 2011. As the first author was planning to conduct fieldwork in the same area later that year, it was discussed that this would be an interesting issue to explore further.

Most of the research for this article took place in an open-air marketplace in the city of Arequipa known as the *Feria* among market vendors where the first author intended to carry out research primarily concerned with occupational health. As more time was spent with participants, patterns regarding how ideas of both bio and ethnomedical systems informed their course of treatment began to be seen. In the process, the researcher became increasingly curious as to why pills are perceived by some as effective for *kharisiri* attacks but not sought as a cure for other folk illnesses that involve spiritual beings. As a country rich in medicinal plants and knowledge about traditional healing, Peru provides an interesting backdrop to explore treatment decision-making in a medically pluralistic context.

### Medical pluralism

Medical pluralism, or the existence of more than one healing system within a given context, is a topic discussed at length in studies of medical anthropology—particularly in economically less developed countries [8,19,20]. These countries are of interest to this field because they have relatively stronger ethnomedicine systems, differing notions of health that may not fit neatly within a biomedical perspective, and sociopolitical conditions that may impact the distribution, access, and quality of biomedicine [19].

Foucault’s concept of “biopower”—a political philosophy whereby the state views individuals as part of populations that must be managed and controlled through the regulation of knowledge and resources—is a lens used in the literature to explore the intersection of bio and ethnomedicine. Bunton and Peterson explain that “the health, illness, death and birth of populations…are directly related to the labor force, economic growth and distribution of wealth” and that biomedicine is a powerful symbol that links one’s body to the authority of state [21]. Through the endorsement and use of biomedicine by governments, the system becomes the officially recognized and therefore dominant medical system in a society. This scientific approach to health however tends to overlook differing health beliefs resulting in unintended consequences [20,21]. For example, after drug shortages in a Venezuelan Amazonian village, participants came to distrust biomedical doctors in a public clinic since they believed they were purposefully withholding treatment [22]. As a result, biomedicine pills were associated with those who hold power and therefore the pills were viewed as powerful themselves.

Related to biomedicine, many examples of power abuse over populations have been documented. In the 1990’s over 200,000 (mostly rural) indigenous women were sterilized in an effort to grow the economy by reducing the population [23]. In the process, healthcare workers—in order to meet strict quotas—lied to the women about the true nature of the procedure, leading many to unknowingly become sterilized. While this campaign’s objective apparently was economic progress, the degree of mistrust that resulted in biomedical procedures and health providers cannot be measured. In another example from a rural Peruvian village, the local health clinic demanded the sick must come to the clinic instead of being treated at home [24]. If they died at home, they were threatened with legal consequences. Villagers were cited as feeling “obliged” to go to the clinic against their will, leading to negative attitudes towards the clinic because it was not their choice to visit. The present study also demonstrates that attitudes of biomedical professionals have a great impact on the decision-making process.

In an attempt to understand motivations regarding health behavior, some have applied models of cognitive decision-making. In the 1950’s, for example, the United State’s government created the Health Belief Model to explain why people did not participate in public health screenings or take medicine as prescribed [25]. As studies in medical pluralism evolving through later decades would show, such models tend to de-emphasize cultural differences, social influence, and larger political issues [19]. Among immigrant groups in Vancouver, for instance, Lin found that treatment patterns varied greatly; Chinese immigrants relied on their family for health decisions whereas those from a European background made more individual decisions about their own care [26]. Lin’s work highlighted how health seeking behavior models tend to assume autonomy in decision-making and also do not take into account social and cultural norms. Crandon’s work in the rural Andes demonstrates how the treatment one uses communicates cultural and social values that are made to form or strengthen social relations [4]. In a similar vein, Fabrega and Silver’s 1970’s study among indigenous Mexicans in a medically pluralistic context stressed that harmonious social relationships, and perceived warmth of the healer, contributed to the treatment decision-making process [27]. From a different perspective, Bruun and Elverdam show how traditional healers change their “explanatory models” of illness when communicating with a client. When explaining an illness, the healer incorporates ideas of both bio and ethnomedicine which has the potential to influence how an illness is understood—thus treatment-seeking patterns should not be understood as static [28]. With such emphasis on social dynamics in the treatment process, it is interesting then to review how those who generally rely on ethnomedicine interpret pharmaceuticals which, could be argued, are the most impersonal of all treatments.

Studies from Asia, Latin America, and Africa—where pharmaceutical distribution increased significantly throughout the second half of the 20^th^ century—have greatly informed this area [20]. A common theme that emerges is how those who have typically relied on ethnomedical treatment perceive pharmaceuticals. Nichter’s work in India revealed that those who identify with Ayurvedic medicine seek biomedicines that posses physical attributes that best fit within their own cultural understandings of health, such as color, and taste [29]. For example, injections are thought to “overheat” the body and thus action must be taken afterwards to “cool” the body. Similarly, Oths’ research in the Northern Peruvian Andes shows how dark colored vitamin tonics were preferred over light colors, since dark colored beer is thought to be curative. Oths also reported that toothpaste was thought to cure toothaches if applied directly to a tooth [11]—a practice that mimics the of the use of curative paste used by traditional bonesetters. Pharmaceuticals have also been noted as being used to prevent harmful effects of pollution and other aspects of a city environment [30].

Specific to conditions that involve spiritual beings, the use of pharmaceutical varies greatly across contexts and illnesses. Logan’s study in urban Mexican areas revealed that none of her 48 participants visited a pharmacy to treat *susto*[31]. Likewise, in Ethiopia, a study of over 400 pharmacy customers produced no data about the buying of pills to treat evil eye and other “possession” illnesses [32]. However, there is some evidence to show use of pharmaceutical products to treat symptoms of folk illnesses. Sikkink discusses how pharmaceutical products in Bolivia are marketed towards symptoms of *susto* but that they are not perceived as capable of curing the underlying cause of *susto*[33]. Similarly, Baer and Bustillo suggest that mothers of children afflicted with *susto* and *mal de ojo* may be receptive to the use of biomedicine as treatment if it’s purpose is framed as alleviating symptoms rather than as a cure for the illness [34].

Pharmaceuticals can also be perceived as being a source of power. Whyte references a study from Zambia whereby women of low socioeconomic status were found to use pharmaceuticals to gain control over their husbands [35]. Moreover, it was thought that foreign medicines were more powerful than locally produced ones because foreign spiritual healers were also perceived as more powerful. Bledsoe and Goubaud also found a particular association between pills and power. Because most pills originate in tightly sealed packaging, they are thought to be powerful since the manufacturers took the time to safely contain them [36]. In this sense, pills may be thought of as way to access powerful healing properties often without subjecting oneself to potentially unpleasant experiences with biomedical health care workers [22].

Indeed there are many factors that can lead to the use of pharmaceuticals in a medically pluralistic setting: convenience due to both physical access and availability without a prescription [19,32,36,37], affordability [29-31,36,38], past experiences [2,19,20,39], or the recommendation from people in one’s social circle or traditional healer^b^[32,40,41]. Because the pharmacist is often he link between pills and those who ingest them, there has been considerable research in this area. Logan suggests that people visit pharmacies when they believe they are knowledgeable enough about their illness to deem what treatment will work [31]. This is similar to ethnomedical processes of healing where the individual, and/or family members, diagnose a condition and then seek – and often self-administer – treatment. In this sense Logan says “a pharmacist’s guidance fits easily into a traditional system of self-treatment that once included only household remedies.” She also found that participants perceive pharmacists as “social equals” who they feel they can consult when they are sick. Likewise Mitchell found that in Jamaica socioeconomic differences between biomedical doctors and participants led to communication issues that affected perceptions of care [42]. On the other hand participants reported that pharmacists better understood their health issues, which often required an understanding of non-biomedical notions of health. Ferguson too documents pharmacists drawing on ethnomedical beliefs when consulting with clients [43].

Others however write about how those who sell pills exploit this knowledge of local health beliefs for economic benefit. In the Sierra Leone, Bledsoe and Goubaud observed that traveling pill sellers described the benefits of the pills differently according to the clients at hand [36]. As noted by Logan, pharmacists too “must please their clients to stay in business” and are “more willing than other practitioners to be amenable to their clients’ ideas about appropriate health care” [31]. Thus, while pharmaceuticals may be relatively impersonal since their use does not necessarily depend on health care providers or physical contact, the role of the pharmacist must be considered when exploring the seeking of pills by those who hold non-biomedical beliefs.

Still, despite considerations given to pharmacists, and cases of local re-interpretations of biomedicines and pharmaceuticals, “relatively little is known, however about how modern prepackaged pharmaceutical products are integrated into alternative medical traditions” [43]. This study will attempt to inform this area, particularly in the Andean context.

### Health & work in the Andes

Many researchers who work in the Andes have noted that natural elements such as wind, cold air, and changes in altitude are perceived to make one vulnerable to illness (see e.g. [4,11,12,39,44]). Such elements are thought to enter the body through porous skin or openings like the mouth, nose, or eyes. Keeping a balance of body fluids is thought to maintain health. To replace lost body fluids in order to restore balance is, however, usually perceived as either impossible or very difficult. In the community of Cuyo, located in Puno—not far from Yunguyo—residents have attempted to restore health by drinking the blood of bats to replace lost blood [45].

As already briefly discussed, it is believed that *Pachamama*, as well as mountain gods known as *apus*, possess qualities that—much like the needs of humans—must be satisfied in order to prevent their anger from causing accidents or illness (see e.g. [1,6-8,12,45-47]). An angry earth, for example, may cause something to happen that startles a person enough to cause their *ánimo* (can be translated as soul, spirit, will, or energy) to leave their body, resulting in *susto.* As conversations with market vendors for this study progressed, it was stated that stray animals and other pests like rats have caused *susto* either to themselves or someone they knew. They also felt that rapidly changing temperatures (in the early morning and at sundown) or exposure to wind could make one vulnerable to malicious spirits, demonstrating the relevance of studying the intersection of folk illnesses and this type of work.

Symptoms of *susto* in children include crying and not sleeping. In adults, symptoms can be “any deviation from normal behavior” [12]. Treatment for *susto* typically includes a soul calling ceremony generally centered on a *mesa*^c^ containing candles, incense, and personal belongings of the suffering person. Ceremonies are ideally led by a *curandero*, often during the silence of the night to eliminate distractions that might prevent the lost soul from finding its way back to the body. The ceremony may be repeated over several nights until symptoms are gone.

Like *susto*, people understand the actions of *kharisiris* as being strongly connected with the spirit world. In the same manner as offerings are given to *Pachamama*, it is thought that offerings can be made to the devil. Those who make offerings to the devil or “talk with the devil” are greatly feared, especially the *kharisiris* who offer blood and fat. *Kharisiris* tend to attack when victims are in a vulnerable state such as while sleeping on a long bus ride, or walking alone late at night [4,15]. The tools used to extract the substances are reported as long needles or a knife, which may or may not leave a mark [15,16]. It has also been reported that *kharisiris* may extract body substances by simply looking at people. It is often said that *kharisiris* can be anyone, which is significant among people who identify as of Quechua and Aymara descent, since they typically view social relationships and reciprocity as central to health and well-being. This distinction will become central to this study’s argument regarding the seeking of biomedical pills for treatment of *kharisiri* attacks.

Understanding why pills are used to cure *kharisiri* attacks can contribute insights into how treatment decisions are made in contexts where multiple options are available. This “insider” perspective may be particularly important in Peru due to the country’s relatively large indigenous population and socioeconomic inequities. To apply perspectives from people working in markets—or in small-scale trade activities on the margins of the formal economy^d^—is of particular relevance in Peru since nearly 71 percent of the working population (who do not participate agriculture) earn their living through unauthorized work [48].

Vending often involves unauthorized and undocumented forms of transaction, and vendors generally do not pay taxes or receive benefits such as sick leave or health insurance. A 2007 WHO report on the protection of workers’ health states that “working conditions appear to be worse for workers employed in small enterprises, the self-employed, and workers in the informal sector. The risks they face are generally of a chronic, long lasting nature, herewith implying negative health consequences” [49]. In a country with one of the most inequitable health systems in the world^e^[50], the perspective of vendors is particularly relevant to help understand how these two systems—bio and ethnomedicine—can be utilized together to lead to more effective use and distribution of health resources. With the present study, it is hoped that the biomedical community will gain an enhanced understanding as to how people with traditional health beliefs decide on a course of treatment in a medically pluralistic environment.

Crandon-Malamud’s work on medical pluralism in the Andes provides a useful framework when attempting to understand how decisions between treatment systems are made, especially for taking into account sociopolitical conditions of marginalized groups. Crandon-Malamud makes sense of treatment choices based largely on the hypothesis that medicines are a “primary” resource, chosen on the basis of the social gain that may result from the treatment choice, that is, the “secondary” resource [4]. In this manner, Crandon-Malamud argues that social relationships are important to one’s livelihood and also relate to preference of treatment choices. By making certain treatment choices, one can either re-enforce or damage relationships that may go on to benefit or harm one’s well-being. This understanding will guide our analysis as we present different cases of participants’ treatment stories. Through these stories we will explore: i) how people perceive pharmaceutical pills and how this understanding may influence their use in relation to folk illnesses that involve spiritual beings; ii) how Andean notions of well-being, including social relationships and the soul, influence treatment choices; and iii) how the historical and sociopolitical context of the *kharisiris* may play a role in the acceptance of pills as treatment for *kharisiri* victims.

## Methods

Data was collected primarily from July to October 2011^f^. The researcher returned for another visit in August 2013, following up with a number of the original key^g^ participants of the study. Most of this time was spent in Peru’s second largest city, Arequipa. The key participants of this study were vendors in the *Feria Altiplano*, a large, traditional open-air market located on the outskirts of the city. The *Feria* consists of vendors who sell mainly vegetables, fruit, vegetables, meat, packaged and prepared food, house wares, clothes, and electronics. In the streets surrounding the market are *ambulantes* (walking vendors) who were also included as participants in this study.

The majority of participants were first- or second-generation Andean migrants from rural parts of the Departments of Puno and Cusco, and most of them were bilingual speakers of Spanish and Aymara or Quechua. First generation participants were typically over 40 years old and tended to follow traditional cultural practices, dress and hairstyle. Because first generation migrants tended to be more knowledgeable about folk illnesses, this group contributed significantly more to this study than those less than 40 years old. Participants under 40 years old typically exhibited more cosmopolitan cultural practices. Pharmacists who contributed to this study also followed cosmopolitan practices, and appeared to have a higher level of formal education than most vendor-participants.

Previously, about half of the vendors who participated in this study worked as domestic servants or as agricultural workers; the others have always been vendors. While vending is a type of work that historically has been common among women in the rural Andes, these professions represent available forms of work for those in the city who lack formal education. Indeed, the researcher observed that participants sometimes had difficulty making correct change for customers, or reading paperwork associated with the governing market association.

Participants were recruited based on convenience, snowball, and purposeful sampling. For the first two weeks of fieldwork, the researcher spent six to ten hours in the *Feria* nearly every day. This allowed the researcher to observe the vendors’ schedules in order to reduce interference with normal activities. During this phase, the researcher began to build relationships by chance with vendors who were receptive to conversation, and many became participants. In several cases, these initial participants introduced the researcher to other vendors with specific areas of expertise—characterized either by the nature of their work, or their knowledge about particular themes the researcher inquired about. The researcher also purposefully sought participants in the *Feria* based on age, gender, and their type of work in order to gain a range of perspectives. As fieldwork continued, the researcher visited the market almost daily to share meals, attend festivals, and accompany vendors on errands or to their homes.

This study is informed by 29 key participants. Twenty-five were female and four were male. The majority of participants were female mainly for the reason that the majority of market traders are female. Three of the 29 key participants worked as *ambulantes* who vended in the street surrounding the market. Participants’ ages ranged from 19-74. As mentioned, most participants however were over the age of 40.

Unstructured interviews took place in the natural setting of the market where participants worked. Appointments to meet with vendors were generally not made since daily activities and were sometimes unpredictable. By simply walking through the market, the researcher was able to identify which established participants were available. The researcher was advised by a local not to use the word *entrevistas* (interviews) when approaching participants but instead to ask if they would like to *conversar* (converse). The researcher emphasized she wanted to know more about their work, and health issues. The interviews generally started by posing an open-ended question to spark a conversation about a particular research theme.

Five main themes were explored: i) occupational health concerns; ii) how health is maintained; iii) preferred treatment methods for any mentioned health issue; iv) experiences with folk illnesses, and; v) how folk illnesses are treated. It should be noted that not all participants contributed data to all themes due to limitations related to availability and hesitation to discuss certain topics. Twelve of the 29 participants contributed data to all themes the researcher sought to explore. The interviews were kept to approximately five to ten minute sessions over the course of several weeks or months depending on the participants’ availability. Sessions were kept short to reduce interference with vending responsibilities. Notes were written either during the time of the conversation or later the same day. An electronic spreadsheet was kept on each participant during fieldwork. The spreadsheets contained basic information regarding participant’s age, where their family migrated from, marital status, aspects of their work, and health. Once fieldwork was complete, data was thematically coded using both predetermined and emergent codes.

Most of the data on biomedicine as well as *susto* is drawn from fieldwork in the *Feria*. Much of the data regarding the treatment of *kharisiri* attacks came from conversations with vendors in the *Feria* who migrated from Yunguyo, where *kharisiris* are said to live in high concentrations. Data on *kharisiris* were collected also during two trips to Yunguyo, where several vendors at the *Feria* have kin or business relationships.

In addition to the 29 key participants at the *Feria*, several secondary informants in Yunguyo—including four pharmacists and approximately eight vendors – also contributed to this study. The four pharmacists were chosen because they happened to be working in each of the four different pharmacies in Yunguyo’s two main squares during the time of data collection. Vendors in Yunguyo who contributed to this study tended to be those who exhibited curiosity towards, and a willingness to converse about the research themes without previous interactions with the researcher due to time limitations in Yunguyo.

Yunguyo is a border town along a popular trade route between Peru and Bolivia in which many inhabitants make their living from associated business activities. Vendors in Arequipa remarked that there are many big houses in Yunguyo and that many people own a car, which are significant symbols of prosperity and success. It was often said that while there may be a set of neighbors who do the same work, one family might be rich and the other not. Many people explained this by noting that one family fed or talked with the devil, and the other did not. Yunguyo is specifically significant for vendors because many travel across this border to obtain goods in Bolivia which are cheaper than in Peru, or alternatively, buy their goods from others who travel this route to obtain goods in bulk and in turn sell them to vendors for resale. The town is also on route to Copacabana, Bolivia, to which thousands of vendors from Peru make pilgrimages every year to make offerings to *Virgen de Candelaria*, *Pachamama* and the *apus*. High in elevation and overlooking Lake Titicaca, the land surrounding Copacabana is thought to be particularly powerful. It is an ancient holy site of the Incas and also the home of the prosperous *Virgen de Candelaria*. Today it is seen as a place to give large offerings once a year for good health and good luck in business.

In addition to trips to Copacabana and Yunguyo, trips to Cusco, Puno, and Bolivia enhanced the understanding of themes related to health and treatment in the Andes. On these trips, countless informal conversations with vendors, *naturalistas* (vendors of natural medicines), two doctors, and approximately twenty additional pharmacists at fifteen different pharmacies helped to inform this study. Pharmacists and doctors were found by chance while walking through areas surrounding the main square in the given city.

## Results and discussion

In Yunguyo, vendors and villagers from around the region convene every Sunday to exchange goods. Because there are few opportunities to obtain items during the week, the market draws thousands of participants, turning a sleepy village into a bustling center for trade. But just as quickly as the market sets up, it comes to an abrupt end. In the late afternoon, traders head back to their homes; this is when the sun starts to fade and the temperatures drop. In the rural Andes, travel by night can be difficult, cold, and dangerous. Vendors face unlit roads and walkways while on alert for thieves who are after unsold goods and profits. At night one is also more apt to be attacked by the *kharisiri*.

José is a vendor of dried *haba* (broad), beans and grains in Yunguyo. He is of middle age and Aymara decent, and is by all accounts a successful vendor. He is well spoken, has studied economics, and has several *ayudantes* (paid helpers), which is a marker of success among vendors. On the researcher’s first attempt at speaking with José (at the suggestion of another vendor), he kindly asked her to return when he was less busy. Several hours later, José enthusiastically, yet quietly, shared his knowledge of *kharisiris*. “There are many ways to treat a *kharisiri* attack” he explained. “Wrap yourself in the hide of a black sheep, eat *cebo* (fat) bought from those who do black magic, or in the pharmacies there are pills.” After the conversations with José, two other vendors at this market confirmed that pharmaceutical pills are being used to cure *kharisiri* attacks. Other contacts in Yunguyo similarly noted the existence of pills that could cure the victims of *kharisiri* attacks. José and the family referred to a specific pharmacy where the pills are being sold, while the others reported that any pharmacy could prescribe pills for *kharisiri* attacks.

To gain another perspective on this issue, nine pharmacies in Puno were visited, five in Arequipa, and four in Yunguyo. The intention was to see if pharmacists could substantiate the extent to which customers ask for medicine to treat *kharisiri* attacks, and if so; do pharmacists sell them pills, and what is typically prescribed? While the responses were varied, four pharmacists in Puno and two in Yunguyo indicated that customers do ask for treatment for attacks. Yet the pharmacists reported major variations in the frequency of such treatment^h^ requests. Some reported this happening several times a day and some once every few months. As such, it is difficult to quantify how often this practice is occurring. However, because the majority of pharmacists said that they are not asked for pills to treat *kharisiri* attacks (especially those in Arequipa), and because the majority of participants reported ethnomedical treatments, it is felt that ethnomedical treatment is indeed more common. We nonetheless find it important to understand people’s rationale behind the use also of pills to cure such attacks.

Pharmacists who did sell pills for *kharisiri* attacks reported prescribing the type of pill based on symptoms. The pharmacists in most of these cases sold a collection of pills: one for fever, one for an upset stomach, one for muscle aches. Sometimes they would give several days’ supply and sometimes just one round of pills, depending on the customer’s situation. It is important to keep in mind that the actual components of these pills are somewhat irrelevant. This study does not seek to determine the biomedical explanation of *kharisiri* attack symptoms, nor what the pills would do physiologically, but instead theorize as to how Andean notions of health, reciprocity, and sociopolitical contexts may affect treatment decisions.

### Pharmaceutical pills

Participants unanimously felt that pharmaceutical pills contain “chemical” properties that are potent and dangerous, especially when ingested regularly. In reference to pills, Rita, a 42-year-old street vendor in Arequipa who moved from the rural highlands in her early 20s, said, “*los quimicos son al instante*” (the chemicals are instant). Others often used the phrase “*en un golpe*” (in one fell swoop) when describing how pills function. Even though participants regarded pills as potentially harmful, the speed at which they work was perceived as an advantage. Nearly all participants who discussed the *kharisiris* commented that treatment must be sought immediately; the longer one waits, the less likely one is to be cured. Therefore, participants’ belief that pills work instantly may contribute to the perception that pills are effective treatment for *kharisiri* attacks.

Additionally, pills seem to offer a level of convenience that may be appealing for busy vendors. Rita said she prefers to treat her illnesses (specifically *la gripe*) with natural remedies like herbal teas. All other market vendors the researcher spoke with also preferred natural treatments. Rita lamented that there is nowhere to prepare natural treatments while she is vending in the streets. Therefore, when she is ill, she resorts to pills because they take effect fast and do not require time or facilities to prepare. By spending considerable time with participants, insight was gained into how they obtain pills, and the social factors that may be at play. As we will demonstrate, social relationships are important to consider in terms of Andean notions of health and well-being, and they are a central theme to discuss when making a distinction between the acceptance of pills over other forms of biomedicine. In the following, we will move on to the issues of social relatedness and *susto*, while continuing to return to questions regarding the perceptions of pills.

### Social relatedness

On two occasions when Rita was suffering from *la gripe*, she showed the researcher pills that she bought from a pharmacy across from her vending post. Rita referred to the pharmacist from whom she always buys as her friend. Further conversation revealed that this pharmacist was also a regular customer of Rita’s, buying orange juice from her several times a day. On one particular occasion, the pharmacist bought homemade cookies from Rita, after Rita had given her a sample for free. Another pharmacist working at the same time was also given a free cookie, but did not buy any. During our conversation, Rita made the distinction that this pharmacist was not her friend and also said that she would not buy pills from her.

The process of creating social relationships in the Andes is generally based on acts of reciprocity, both with the natural world and with other people. On a daily basis, such acts involve the exchange, sharing, or offering of food, drink, and coca leaves. To refuse to partake in meals or accept food when offered sends the message that one does not wish to enter into social relations with the given person or group. In Rita’s case, she may have felt socially rejected by the pharmacist who refused to buy her cookies. Her preference for the pharmacist she considers her friend may then be indicative of the influence of social relationships in the rationale of treatment choice. If this is true, then the ability to form reciprocal relationships with nearby pharmacists—who are vendors themselves—may be important to consider when discussing why some people use pills to treat *kharisiri* attacks. As we discuss in the following sections, this social bond may be lacking in other encounters with biomedicine, such as when seeking treatment from a doctor.

Rita was not the only participant to regard a pharmacist positively. A fruit vendor also talked about the “nice woman” who “always” helped him at the pharmacy. Another participant offered to introduce the researcher to her dentist^i^ without an appointment just as one may show up at a pharmacy. In contrast, participants (with one exception) did not make reference to a particular biomedical doctor. While this may be due to the many barriers to accessing the facilities where doctors practice, such as time and money constraints, it may also be a result of the one-way economic exchanges with staff in a way that is devoid of reciprocity. For example, people said that in hospitals one must pay to be seen in a waiting room before receiving treatment. Once admitted, it is expected that patients bring gifts for nurses, such as chocolate, or they will not care for you. Pharmacists, on the other hand, do not need to be “bribed.” The price of the pills is agreed upon upfront by the pharmacist and the customer in much the same way a vendor may discuss a price with their customer. An additional dimension to consider is that of the relationship to a regular client. It is common practice that a regular customer of a vendor will get special treatment, often being given a bit extra of what the vendor is selling as a symbol of appreciation for the regular business. By regularly visiting the same pharmacist, vendors themselves may expect or benefit from similar patterns of exchanges, particularly in this context where the pharmaceutical industry is relatively unregulated and prescriptions are often not needed [38,51].

Beyond potential benefits of being a regular customer, the informality that often exists within the buying and selling of pills mimics the unregulated selling of goods in and around the *Feria*, and certainly in most other open markets in the Andes. The vending of goods—the authenticity and legality of which may be questionable—positions pharmacists and vendors in a similar social occupational category, which may allow vendors to identify with pharmacists on a socioeconomic level. As illustrated in Logan’s study, clients who relate to pharmacists tend to prefer pharmacists to doctors. Combined with a mutual negotiation of their relationship through the goods they sell, these dimensions contribute in building relations of trust.

The importance of establishing social relations with health professionals should not be overlooked. Wayland and Crowder have explored peoples’ attitudes towards community health workers in an Andean community in Bolivia [52]. Growth in the community prevented health workers from being able to make as many visits to communities as normal. Community members’ attitudes revealed that they perceived community health workers as “strangers” who were just like “anyone else on the street.” Resulting evidence showed that their health advice was rejected and unwanted. In contrast, Rita’s friendship with the pharmacist may have increased the degree to which she was willing to discuss her health and be receptive to treatment recommendations. Such an example suggests a degree of trust that must precede relationships with health professionals.

To illustrate this argument further, it is worth discussing more in depth participants’ attitudes towards biomedical doctors. In Laura’s story below, she seeks treatment for her young daughter from both a biomedical doctor and a *curandera*. While the treatments were for the same illness and occurred in a very close timeframe, she attributed the healing to that of the *curandera*, not the biomedical doctor. As we argue, the contrast of the social relationships involved in the *curandera’s* traditional healing and the doctor’s treatment, and the participation in the healing ceremony by Laura’s family, appear to have influenced Laura’s reasoning. This case will also highlight how *susto* is conceptualized differently than *kharisiri* attacks, which will be an important factor in how the acceptance of pills for *kharisiri* treatment is analyzed further.

### Susto and Mal de Ojo

When Laura’s two-year-old daughter fell ill with symptoms of vomiting, not sleeping, and crying more than usual, she brought her to the hospital. While she was waiting to see a doctor, she discussed her daughter’s symptoms with a woman in the waiting room. The woman felt Laura’s daughter was suffering from *susto*^j^ and gave her the contact information of a *curandera*. Laura proceeded to see the doctor, whom she said “did nothing” except prescribe a liquid medicine. Laura gave her daughter the medicine, which she also said “did nothing.” Indeed, as Finkler argues from her work in Mexico, the perceived failure of a treatment from one system is not uncommon in leading to a diagnosis of a spiritual cause through process of elimination [53]. Later in the day Laura contacted the *curandera* whom she was recommended at the hospital and that night a soul calling ceremony was performed in Laura’s house.

The *curandera* arrived around nine o’clock and all the family members who lived in the house gathered in the same room. Together they performed rituals with herbs and incense and items that belonged to Laura’s daughter. The use of personal items, such as clothing or in this case a doll, is regarded as important in these treatments so that the lost soul will recognize where to return. The ceremony lasted until two in the morning and Laura said her daughter was healthy again after a few days. Even though Laura also gave the biomedicine to her daughter, she credited her recovery to the soul calling ceremony.

Laura, like many participants, emphasized that soul calling ceremonies are typically held on Tuesdays and Fridays, which are considered the best healing days. If a ceremony is not believed to be effective, it may be repeated on the following healing day, typically up to a total of three times. Therefore, a week can pass before a “round” of treatment can be completed, and thus evaluated for effectiveness. Biomedicine, on the other hand, as mentioned, is expected to work immediately. Laura did indeed give her daughter the medicine prescribed by the doctor but also commented that her daughter was better a few days after the soul calling ceremony. Perhaps because her daughter was not better immediately after taking the biomedicine, she believed the medicine was not responsible for curing her daughter. Since a week can pass before a soul calling treatment can be completed, the several days’ delay of her daughter’s healing may be why Laura emphasized the role of the healing to restoring health over the biomedicine, which is thought to work quickly.

As in the case of Rita and her pharmacist, social relations may also be at play in Laura’s case. The time the *curandera* spent with the sick girl may have contributed to the perceived efficacy of her treatment. The *curandera* spent five hours with Laura’s daughter, which is likely to be much more time than the child spent with the biomedical doctor in the hospital. This length of time may give the impression that the *curandera* “did something” whereas the doctor “did nothing.” The time spent with the child would allow the *curandera* to get to know Laura’s daughter in a more holistic sense. Bastien quotes Abraham Maríaca’s statement that “Andeans perceive the doctor without a heart because he charges a lot, treats them like a machine and keeps apart from them. The scientific method brings out these features in the doctor. On the other hand, Andeans love the *curanderos* who massage, console and communicate with them” [39]. Children are believed to be especially vulnerable to soul loss and other folk illnesses since their soul is not yet considered strong. A biomedical doctor may be perceived as not holding these same beliefs and therefore not able to treat a child’s illness in the same holistic way. The fact that the *curandera* treated the child at home may also have contributed to the efficacy of the traditional treatment in terms of social relations, as it involved the whole family.

As outlined earlier, Crandon-Malamud suggests that medical treatment can be considered a “primary resource” and through its use, one acquires “secondary” resources. Secondary resources are generally socially or politically motivated. One may choose a treatment to align oneself with one’s community or as a statement of one’s beliefs and values. Participants, largely due to discrimination, costs, and long waiting times, did not generally regard hospitals and biomedical doctors positively. In contrast, many participants told positive stories of traditional healings and even offered to introduce the researcher to *curanderos* they knew. In terms of resources involved in the treatment, Laura and her family were active participants in the healing process, which may be an important factor in why credit was given to the c*urandera*. Laura depends on her family to run a successful banana stall. Her husband is in charge of acquiring and restocking the bananas she sells every day. Laura’s mother cares for her children six days a week while Laura vends, and one day a week her mother vends so Laura can have a day off. Without the help of her family she would likely have to work many more hours and pay for bananas to be delivered. According to Crandon-Malamud, then, it may be that Laura credited the traditional healing as being effective as a way in which to maintain positive ties with her family that support her economic success. Or in other words, by validating the traditional healing (primary resource), she also validated her family’s time and effort, which would work to promote her economic well-being (secondary resource).

This case also demonstrates how different ideas of temporality may influence understandings of treatment effectiveness. For instance, unlike *kharisiri* attacks that demand immediate treatment, it seems the urgency for treating *susto* is less, making slower-working traditional treatments appropriate. Furthermore, because immediate treatment is not required, healing *susto* can wait until vendors (who mentioned not even being able to prepare traditional teas in the market) are free from work, since healing ceremonies occur late at night. The immediacy of treatment that *kharisiri* attacks require would not be conducive to work if traditional methods were to be relied on. Laura’s case also shows how important social relations are—not just with the person providing treatment but also with those participating in the treatment.

When participants were asked about treating *susto* with biomedical pills, the answer was unanimously that they were not effective. *Naturalistas* in the market who were knowledgeable about healing also said that pills could not cure *susto* and shared the details of healing ceremonies and the materials needed. Vendors repeated the same advice with little variation. The reaction was the same for *mal de ojo* (evil eye).

*Mal de ojo* was nearly always spoken about in reference to children, as children are thought to have weaker souls than adults. It was said that if an ill-meaning adult looked at a (usually particularly good-looking) child too long, the child’s soul could be taken over by the stronger adult’s soul, which was thought to be evil. The child, it was said, would suffer from many of the same symptoms as *susto* (not sleeping, irritability, fever). Participants described treatment that was intended to “draw out” the evil soul by passing a fresh, uncracked egg over the child’s body, as eggs are thought to have absorbing qualities [54]. The egg would then be broken into a glass and examined for any abnormalities. A broken yolk, strange markings, or a somewhat solidified egg would indicate a presence of evil and also that it had been successfully removed from the child [55].

Like *susto*, *mal de ojo* is characterized by a disruption of the soul, which is often thought to be a result of failing to uphold reciprocal relations with *Pachamama* or those within one’s social circle. The understanding of the soul’s involvement in an illness seems to determine whether an illness is understood as bio or ethnomedical in origin. When the researcher asked Rita if a *curandero* could treat *la gripe*, which she would otherwise go to the pharmacy to buy pills to treat, she said while laughing, “*no, la gripe no tiene nada que ver con el alma*” (no, the flu has nothing to do with the soul). An important distinction of the *kharisiri*, which we were able to draw from participants’ descriptions, is that the *kharisiri* attacks—like *la gripe*—have nothing to do with the soul.

### Kharisiris

According to scholars, the *kharisiris* appeared in Aymara history around the time the Spanish arrived [4,14,39], and there has since been an understanding of the *kharisiri* as being a fat and blood snatching foreigner, or stranger^k^. The loss of fat at the hand of foreigners is, according to Bastien, symbolic of social and political inequities, whereby its loss represents further abuse and exploitation [2]. Other scholars have interpreted the *kharisiri* as a personification of violence in the relationship between highland and the capital, and between Peru and the United States [55], as a response to modernization [56] and as involving an epistemology of race [16,17]. Weismantel explores the *pishtaco* as an indigenous expression of racial violence, and underlines how his dreadful deeds depict whiteness and masculinity as powerful and threatening. While the *kharisiri* may thus illustrate how whiteness and masculinity is understood from an Andean perspective, Canessa explores how the *kharisiri* can also illuminate how “indianness” is understood by Indians. As we will return to, he does so by examining the cultural meanings of fat. *Kharisiris* are thought to be for instance Catholic priests, politicians, engineers, or biomedical doctors. These positions are either associated with knowledge that originated abroad, or access to power and resources that have worked to exploit people in the Andes. Indeed, several participants stated their belief that the bodily substances the *kharisiri* takes are often sold to foreign countries (the United States and Europe were both mentioned) for use in manufacturing that results in “big profit.” Participants often remarked that the extracted fat was exported to other countries where it is used to make consumer goods such as soap and candles, or even used as a lubricant for machines.

This perception that the foreign market profits from fat is referenced by many researchers in the Andes. Crandon-Malamud too writes about how fat was used to create fancy soap to export and to sell to tourists. She also mentions that North Americans used the extracted fat to power their electricity [4]. Weismantel describes an artistic display set in the 1960’s by an artist in Ayacucho whereby human fat is used to grease engines of airplanes and “modern” machinery [16]. More recently, in the 1980’s, it was rumored that fat was used by government officials to pay off Peru’s national debt [57]. According to Crandon-Malamud, *kharisiris* have later been associated with the use of fat for the production of cosmetics and medicines at factories or pharmacies, and it was believed that “the materials to extract it [fat] could be bought clandestinely in pharmacies in La Paz” [4]. This understanding of fat and exploitation is essential to our arguments surrounding the use of pills as treatment.

In *Fear and Loathing on the Kharisiri Trail: Alterity and Identity in the Andes,* Canessa notes: “Other illnesses respond to treatment through divination and offerings to the earth spirits, but *kharisiri* attacks are outside the relationship people have with the tellurian spirits and therefore the spirits cannot be invoked to cure the victim” [17]. The two cures Canessa observed in his research were 1) to kill the *kharisiri*, or 2) to buy “back” what was taken, generally fat that his participants said could be found in pharmacies. Canessa however makes no mention of pills.

The connection Canessa encountered between fat and pharmacies is perhaps not as peculiar as it may at first appear, since people seemed to make a connection between pharmacies, foreign countries, and exploitation. As noted by Crandon-Malamud, there is also a belief that biomedical pills are made from human fat, stolen by *kharisiris*. The participants in our study often associated pills with other countries and often asked the researcher to mail vitamins to them when she retuned to the United States. Participants stressed that pills manufactured abroad (the United States and Europe were mentioned again) were of better quality because education, science, and technology are more “advanced” in these foreign countries. Additionally, it was understood among participants that several of the big chain pharmacies in Peru are owned by enterprises in neighboring South American countries^l^. The sentiment that foreigners exploit the people and land of Peru was not uncommon. Informants sometimes discussed with the researcher how other countries, like Chile, take Peru’s rich natural resources (coal and oil, for example) without giving Peru anything in return. This, informants explained, is a reason why Peru remains economically poor. Since the *kharisiri* is perceived as a foreign and exploitative figure, it is perhaps significant that some people seek pills from an industry that embodies these same characteristics. Drawing on Foucault’s notion of biopower, Miles for instance notes that “because medicines are meant to heal, those who control their production, and distribution garner a measure of social power…pharmaceuticals, in particular, carry powerful associations with science and technology, and consequently, those who control their distribution are symbolically associated with the power of advanced technology” [41].

Furthermore, as explained previously, participants believe pills as fast working but also dangerous because of their “powerful” properties. These potent pills may be perceived to be effective in combating an illness that is caused by powerful oppressors—such as foreigners or those who hold political power—who have access to fat-removing technology and perhaps also connections to sell fat for profit abroad [4]. Keeping in mind that participants perceived healing methods to be quite literal interpretations based on the source of illness (appeasing *Pachamama* to restore reciprocity, calling the soul back, or drawing out the evil that entered the body in the case of *mal de ojo*), swallowing a pill that represents an industry steeped in foreignness may embody curative elements for *kharasiri* attacks. On the one hand this suggests a dependency on the pharmaceutical industry among people who may be perceived as being exploited by it, but on the other hand, pills may also offer a cheaper alternative to traditional cures such as buying fat, which is particularly expensive, and as Canessa notes, is a “double exploitation” [17].

Fat is associated with life force, and is seen to be something of a protective factor against the hard physical labor and rapidly changing air temperatures that characterize life in the Andes, which are conditions that vendors are particularly exposed to. When the researcher asked participants how they stay healthy enough to work, they nearly always replied, “I eat a lot.” Canessa points out that *kharisiris* are thought not to attack children or the elderly but only middle-aged adults who are “most clearly engaged in production and reproduction.” Thus he makes the point that when those involved in economic activities are attacked, they—and their families who rely on them—are more likely to remain oppressed due to reduced economic productivity caused by illness or death. Seeking pills for treatment of a *kharisiri* attack may be an attractive alternative for vendors because they do not represent a significant cost and also because a pill’s effectiveness is expected to be felt immediately, allowing vendors to continue to work and be economically productive.

The economic underpinnings of the *kharisiri* are perhaps even more clearly illustrated when seen in the perspective of a broader historical context. As Crandon-Malamud documents, the ways in which the *kharisiri* is described tend to change based on the given sociopolitical climate. Based on research in a small Bolivian village, Crandon-Malamud writes that up until the 1950s the *kharisiri* was “universally the image of a dead Franciscan monk” who gave victims’ fat to a bishop to make holy oil [4]. In the following decades the *kharisiri* was associated with North Americans, after the community had contact with foreign aid programs that were interpreted “as an attempt by the United States to practice genocide for imperialist gain.” In the late 1970s the *kharisiri* was associated with the Bolivian elite and with “any *mestizo* who participated in the trade of human kidney fat.” Crandon-Malamud’s point is that the *kharisiri’s* identity changes according to who is perceived as the oppressor, and furthermore stresses that society’s social structure may be linked to one’s understanding of health. She goes on to mention that in the late 1970s, there was a period of reform in the community she studied when a health clinic was built and distribution of land became more equitable. At the same time, it became possible to cure a *kharisiri* attack, which previously was perceived as deadly. It may be that as the understanding of the *kharisiri* evolves so too do the ideas of how to cure its victims.

Furthermore, the methods and tools *kharisiris* are thought to use for removing bodily substances are also changing as technology progresses. Fernández Juárez writes that the *kharisiri* used to be perceived as “cutting” victims with their own knife [14]. Now, he says, modern tools are used in extraction, such as cameras, tape recorders, and syringes. Participants in this study almost always mentioned syringes or needles. José even mentioned the use of lasers, which he explained could be used from afar. The evolving nature of the *kharisiri* in the context of an ever-changing society may perhaps contribute to the variety of treatments that have been noted throughout history—and why more may continue to be added. Historically, remedies for treating a *kharisiri* attack included several traditional treatments like eating fat, drinking blood, wrapping one’s stomach with the hide of a black sheep, and drinking various homemade brews. Among participants there was no single cure that was unanimously recommended. In contrast, participants mentioned only one treatment for *susto* and *mal de ojo,* which are both connected to the soul and/or social relations and are conditions that have been deeply embedded in Andean culture. The *kharisiri,* on the other hand, is a relatively new figure, appearing in history around the time of the Spanish arrival.

Another reason why pills may be accepted as a form of treatment is that the soul and social reciprocity do not seem to play a role in how the illness is understood. As discussed, *kharisiris* are associated with strangers, implying a lack of social reciprocity leading up to the illness and therefore helping to explain the lack of acts of reciprocity involved in curing the illness. Participants reported that the *kharisiri* “can be anyone”, and thus attacks do not seem to have roots in social relations. Therefore treating an attack does not appear to have any direct outcomes other than bettering one’s health. The lack of social involvement in the origins of the attack may make the use of biomedical pills—which are less personal than all-night healing ceremonies—more likely to be perceived as an appropriate treatment. This is further evidenced by the many cures for *kharisiri* attacks participants listed, which all involved obtaining various substances and usually ingesting them to “replace” what was lost. There seemed to be little to no emphasis on relying on family members or friends to obtain the healing substances. The emphasis was instead on ingesting the substances rather than on the *process* of healing as we saw with the all-night soul calling ceremony, and the method for treating *mal de ojo*. Additionally, in cases when a soul was returned to the body, participants said they “just felt it.” A soul however, cannot be seen in the same physical sense that fat and blood can be. Therefore, ingesting a pill that can be seen and felt may be perceived as a way to “replace” other physical elements taken from the body. Indeed, considering the previously mentioned association between biomedical pills and human fat, the use of pills to cure *kharisiri* attacks may be a result of the view that pills contain similar substances to that of fat, only in another form.

It is important not to associate the acceptance of pills with the acceptance of all biomedicine to cure *kharisiri* attacks. Referring to the *kharisiri*, participants commonly said “*los medicos no creen*” (the doctors don’t believe), and if you go to the hospital to be treated for an attack, the doctors will give you an injection and you will die almost immediately. Why is it that injections are perceived to kill but pills are perceived to heal? There are likely to be several explanations to consider. It may be that people believe injecting the body with foreign substances (especially liquid injections) may throw off the “balance” of body fluids necessary for health that is understood under Andean humoral health beliefs. In contrast to the sentiment that doctors “don’t believe”, the seeking of pills may also be explained in part due to pharmacist’s own belief in, or validation of, *kharisiri* attacks.

Juanita, who works as a nurse during the week and a pharmacist on the weekends in Yunguyo, said that on Saturdays and Sundays “*harta gente*” (many people) come to ask her for pills to treat *kharisiri* attacks. She stressed that her husband—a medical doctor—thinks that people are ignorant to believe in the *kharisiri* “myth.” Juanita said that after fifteen years of working at the pharmacy, she now “believes in the *kharisiri* for her client’s sake.” Based on symptoms, Juanita said she typically prescribes these customers several rounds (the number of which depending on what her clients can afford at that point in time) of three different pills to be taken three times daily with meals. She remarked that instructions to take pills with meals helps the clients, the majority of who are vendors, remember to take the pills at regular intervals because she knows that they place an emphasis on eating regularly but are not always aware of the time. It is difficult to say if customers come to Juanita because they have heard about her treatment through others, or if they arrive by chance. Regardless, it appears that by listening to her clients without exhibiting judgment, she fosters an environment in which clients feel comfortable to express their health concerns, as they understand them. While on the one hand this process personalizes the relative impersonal nature of a *kharisiri* attack, the treatment process at the pharmacy still lacks the physical touch and time investment that characterizes the work of traditional healers. In her interactions with clients, she nonetheless validated their beliefs in a way that made clients trust that she knew what pills could treat their illness.

The lack of physical contact in pharmacist interactions may also be an advantage because the situation does not appear to give pharmacists the ability to take any more substances from an already weakened body. Fernández Juárez writes that doctors are commonly viewed as “allies” of the *kharisiri* who take fat from patients to profit from [14]. According to Bastien, “Bolivian doctors are skilled surgeons and so are referred to as “*kharisiris*” (cutters) by many rural Andeans.” [2]. In a hospital, where injections are given, one would be surrounded by those with the know-how to extract fat (doctors), who may be perceived as the source of harm in the first place. If a *kharisiri* victim believes this to be so, then the doctor may have motivation to kill the patient to make a bigger offering to the devil, and thus benefit from a larger payoff. With this mindset, seeking treatment in a hospital seems counterintuitive. Drawing from participants’ positive and reciprocal exchanges with pharmacists, it may be that a pharmacist’s ability to relate to clients and their beliefs increases trust, and therefore effectiveness, of biomedical pills over other biomedical options.

## Conclusion

As we have illustrated, biomedical pills are sought to treat *kharisiri* attacks but not for other folk illnesses that involve spiritual beings. This may be because other folk illnesses, such as *susto* and *mal de ojo*, have stronger social and spiritual causations, involving one’s social relationships and a notion of soul (*ánimo*). *Kharisiri* attacks, on the other hand, have roots in broader socioeconomic influences, and are less personal in terms of one’s social network. Such attacks are not considered to affect the soul but primarily the organs and fluids of the body. Furthermore, attacks seem to be relatively random, lacking correlation with social reciprocity and a balance—or the lack of such—with *Pachamama. Kharisiris* are, rather, associated with strangers, implying a lack of social relationship or reciprocity leading up to the illness. This may help explain why acts of reciprocity are not considered important in curing the illness. The “impersonal” nature of the *kharisiri* attack may allow one to consider forms of treatment that are not dependent on social motivations or spiritual causation, or secondary resources in Crandon-Malamud’s terms. Laura’s case of diagnosing and curing her young daughter of *susto* demonstrates the complexity of social relationships in illness and treatment among people in the Andes. It wasn’t until soul loss was considered as the source of illness that Laura discussed her family members as playing a role in restoring her daughter’s health. If she had attributed the healing to the biomedical doctor, it would mean dismissing her family’s efforts and risking damage to her reciprocal relations with the people who support her in business.

As we have demonstrated, perceptions of biomedicine, and more specifically biomedical pills, appear to play a role in their use in treating *kharisiri* attacks. Pills are perceived as fast working, which is an advantage for victims who are more likely to recover if they receive treatment quickly. For victims whose physical properties of fat and blood were taken, ingesting a pill that can be seen and touched may in a sense be an attempt to replace missing elements of one’s physical body. To that end, because pharmaceutical pills are associated with other more technologically “advanced” countries, their use may be seen as an appropriate treatment for illnesses caused by the *kharisiri*—as the *kharisiri* too is rooted in foreignness.

Lastly, an important explanation for why treatments of *kharisiri* attacks are sought from biomedicine may be found in the attitudes of those in a position to provide health care. In Yunguyo, for instance, there were cases of biomedical health professionals who reported they believe in *kharisiris* for their patients’ sake. Given the significance demonstrated by participants of their relationship with those from whom they seek health advice or care, it may be that *kharisiri* victims feel comfortable confiding in a pharmacist who recognizes the harm that *kharisiris* may cause.

The acceptance of biomedicine in the treatment of *kharisiri* attacks should not be taken as a sign of moving away from traditional systems, but instead highlights the capacity for treatment systems to work simultaneously in attempts to fulfill health needs. From the stories told in this article, we hope to have shown that it is too narrow to perceive folk illnesses as a single category; doing so is not sufficient when exploring rationalization of treatment choices. Instead, the underlying cause of such illnesses, whether it be social, spiritual, or a reaction to changing economic or political conditions, may greatly enhance understanding of such ailments and their treatment. Tolerance and respect for those suffering from these conditions may in some circumstances promote the seeking of care across medical systems. If this is so, then drawing on the strengths of multiple medical systems may lead to more appropriate treatment and better health outcomes.

## Endnotes

^a^Biomedical interpretations of the symptoms are inconclusive. They have been traced to Chagas disease [2], cholera, and [15] tuberculosis.

^b^Given the amount of literature on traditional healers who give, prescribe, or advise clients to seek pharmaceutical pills, it should be noted that no participants mentioned traditional healers in discussions regarding pills and *kharisiris*.

^c^Literally translating to “table”, a *mesa* (also sometimes referred to or spelled as *misa*) is a ceremony held in houses that centers around items perceived as suitable to feed the earth, such as llama fat, animal fetuses, herbs, and alcohol. It is held both for healing purposes and to make an offering to maintain general equilibrium and well-being—especially during agricultural months when *Pachamama* is most hungry, such as February and August.

^d^These forms of work have often been addressed as “informal” and belonging to the “informal economy” or “informal sector” (see e.g. [58,59]). These terms may be problematic, however, in that they assume a separation, and understatement, of the ways in which these economic activities intersect with the economy more generally. This study will mainly use terms like “unauthorized” to describe work in this regard.

^e^In 1991 the public health system received just 15 percent of the funding it received in 1980 [23].

^f^Prior to fieldwork, ethical approval to carry out this research was obtained from the Department of Health Promotion at the University of Bergen (UoB) and from the Norwegian Social Science Data Services (NSD) in May 2010 (Project Number: 27219). The study was also carried out in affiliation with the Social Science Department at Pontificia Universidad Católica del Perú (PUCP).

^g^Key participant defined as a participant who the researcher was able to converse with more than once about one or more research themes over the course of fieldwork.

^h^In Arequipa, none of the pharmacists mentioned that clients came seeking treatment for *kharisiri* attacks, but they did report clients looking to treat symptoms that characterize *susto.*

^i^Participants seemed to regard dentists more positively than medical doctors.

^j^When asked what would have caused her daughter to lose her soul, Laura said that it must have been from the time a rat in the market frightened her while she was pregnant.

^k^It should be stated that *kharisiris* are not necessarily strangers or foreigners. The second author observed a case in which a victim of a *kharisiri* attack suspected his sister’s partner to be responsible*.*

^l^According to a WHO report, the counterfeit pharmaceutical trade in Peru is a $66 million per year industry [see: http://www.who.int/medicines/services/counterfeit/impact/TheNewEstimatesCounterfeit.pdf].

## Competing interests

The authors declare they have no competing interests.

## Authors’ contributions

AB designed and carried out the study with the guidance of CØ. AB analyzed the data and wrote the text. CØ contributed with the original idea for the research theme, as well as analytical and editorial input. CØ also contributed with regional experience based on extensive fieldwork in the area since 1997. The photograph was taken by AB. Both authors read and approved the final manuscript.

## Authors’ information

Amy Blaisdell has a master’s degree in health promotion from the University of Bergen. She studied Communication and Anthropology as an undergraduate at the University of New Hampshire. Her research interests include health and gender in the context of unauthorized work, medical pluralism, and health inequities.

Cecilie Vindal Ødegaard is associate professor at the Department of Social Anthropology University of Bergen. She has a doctoral degree in social anthropology, and is author of the monograph *Mobility, Markets and Indigenous Socialities: Contemporary Migration in the Peruvian Andes* (Ashgate 2010). Based on several periods of fieldwork in Peru since 1997, her work has been published in *Journal of the Royal Anthropological Institute*, *Ethnos Journal of Anthropology*, and *Forum for Development Studies* among other journals. Her research interests include questions of indigeneities, kinship, gender, animism; mobility and urbanization; territoriality, state, work and informal economies. Ødegaard is also involved as a researcher in two joint research projects funded by the Norwegian Research Council, titled “Localizing Globalization. Gendered Transformations of Work”, and “Contested Powers: Towards a Political Anthropology of Energy in Latin America.”
